# Limited Diagnostic Value of miRNAs in Early Trauma-Induced Liver Injury: Only miRNA-122 Emerges as a Late-Phase Marker

**DOI:** 10.3390/diagnostics15020179

**Published:** 2025-01-14

**Authors:** Jason-Alexander Hörauf, Amit Singh, Maika Voth, Hamed Moheimani, Cora Rebecca Schindler, Borna Relja, Liudmila Leppik, Ingo Marzi, Dirk Henrich

**Affiliations:** 1 Department of Trauma Surgery and Orthopedics, University Hospital Frankfurt, Goethe University Frankfurt, 60596 Frankfurt am Main, Germany; 2 Pittsburgh Trauma Research Center, Division of Trauma and Acute Care Surgery, Pittsburgh, PA 15213, USA; 3 Department of Trauma, Hand, Plastic and Reconstructive Surgery, Translational and Experimental Trauma Research, University Hospital Ulm, Ulm University, 89081 Ulm, Germany

**Keywords:** liver injury, miRNA, trauma, inflammation, biomarker, L-FABP

## Abstract

**Background/Objectives**: Liver injury is common after abdominal trauma. However, the established biomarkers of liver injury, such as alanine aminotransferase (ALT) and aspartate aminotransferase (AST), lack accuracy. This study investigates whether specific liver-related microRNAs (miRNAs) are released into the circulation in trauma patients with liver injury and whether they can indicate liver damage in the early phase after major trauma. **Methods**: A retrospective analysis of prospectively collected data and blood samples from 26 trauma patients was conducted. The levels of miRNA-21-5p, -122-5p, -191-5p, -192-3p, and -212-3p were measured in patients with computed tomography-confirmed liver trauma (LT group, *n* = 12) and polytrauma patients without liver trauma (PT group, *n* = 14) upon emergency room (ER) admission, and 24 and 48 h after trauma. Additionally, liver-type fatty acid binding protein (L-FABP) was measured, as it has recently been discussed in the context of abdominal trauma. **Results**: Only miRNA-122-5p showed a significant increase in the LT group compared to the PT group, but only at the 48 h time point (*p* = 0.032). Conversely, L-FABP (*p* = 0.018) and ALT (*p* = 0.05) were significantly elevated in the LT group compared to the PT group at the time of ER admission. There was a moderate correlation between miRNA-122-5p and AIS_Abdomen_ (*p* = 0.056) and transfused red blood cell concentrates (*p* = 0.055). L-FABP correlated strongly with the ALT levels (*p* = 0.0009) and the length of stay in the ICU (*p* = 0.0086). **Conclusions**: In this study, the liver-specific miRNA-122-5p did not effectively indicate liver injury in the early acute post-traumatic phase. Future research with a large sample size should investigate whether other miRNAs can more accurately predict liver injury and the extent of hepatocellular injury, particularly in the acute post-traumatic phase.

## 1. Introduction

Major trauma remains the leading cause of death in young adults under the age of 45 [1]. Following abdominal trauma, the liver is the most frequently injured organ [2]. The liver is the largest solid organ in the body and plays a crucial role in several physiological processes, including macronutrient metabolism, regulation of blood volume, support of the immune system, and endocrine control of growth signaling pathways, among others [3]. While minor liver injury accounts for the majority (80–90% grade I and II) of liver trauma cases, severe liver injury is the leading cause of death in abdominal trauma, with a mortality rate of 10–15% [4].

Mortality is not only due to direct liver trauma but also to post-traumatic liver dysfunction/failure. Acute liver failure can occur in 5–10% of polytrauma or hemorrhagic shock cases, triggered by perfusion disturbances leading to hypoxia, barrier failure, or bacterial translocation [5]. To properly monitor liver function after trauma, the serum concentrations of the liver transaminases aspartate aminotransferase (AST), alanine aminotransferase (ALT), and γ-glutamyltransferase (GGT) can be determined. Originally defined as specific markers of hepatocellular damage, AST and ALT levels can be elevated due to extrahepatic disorders such as hemolysis and muscle tissue injuries, and thus lack specificity as markers of liver injury [6,7].

A promising liver-specific biomarker is the liver fatty acid binding protein (L-FABP), which belongs to the FABP family of small intracellular or plasma membrane-resident proteins [8]. FABPs have high tissue concentrations at low normal plasma concentrations and are released in a sensitive and stable manner upon tissue injury, making them suitable as biochemical markers [9]. Previous trials have shown that L-FABP is useful in indicating trauma-related abdominal injury [10,11]. Furthermore, in a recent study, we have shown that L-FABP is significantly elevated not only in polytraumatized patients with liver injury, but also in traumatic kidney injury, thus lacking specificity for liver injury [12].

Over the past two decades, microRNAs (miRNAs), short, non-coding RNA molecules approximately 18 to 23 nucleotides in length, have attracted increasing interest as sensitive and specific biomarkers for numerous diseases, with key roles in gene regulation by mediating mRNA degradation and regulating transcription and translation [13]. Due to their evolutionary conservation, the ease of extraction via minimally invasive methods (e.g., blood sampling), high stability in biofluids compared to protein biomarkers, and the availability of simple, quantitative, and highly sensitive assays such as polymerase chain reaction (PCR), miRNAs hold great promise as more specific and sensitive biomarkers [13].

As miRNA-122 is highly enriched in liver tissue and is specifically expressed by hepatocytes, where it accounts for 50–70% of the total miRNA content in the liver, it is a viable candidate as a liver-specific biomarker [14]. Studies in humans and rodents have shown that circulating miR-122 levels increase in response to drug-induced hepatocellular injury and can be detected earlier than ALT [15,16]. Aberrant miRNA-122 expression has also been associated with various liver diseases, including viral hepatitis, alcoholic and non-alcoholic fatty liver disease, and hepatocellular carcinoma, highlighting its pivotal and pleiotropic role in hepatic function modulation [17,18,19]. However, research into the trauma-induced alterations of miRNA-122 in the context of liver trauma remains limited.

This study aims to investigate whether liver-related miRNAs (miRNA-21, -122, -191, -192, and -212) are released in increased amounts into the circulation in trauma patients with liver injuries, thereby evaluating their potential as indicators of liver injury in the context of polytrauma.

## 2. Materials and Methods

### 2.1. Ethics

This study was conducted at the University Hospital Frankfurt with the approval of the institutional ethics committee (89/19, 375/14) in compliance with the Declaration of Helsinki and adhering to the STROBE guidelines [20]. All the subjects provided written informed consent, either personally or through a legal representative, and all the clinical data were prospectively documented within both the German Trauma Registry [21] and the national Serum Biobank [22].

### 2.2. Patients

From 1 January 2018, to 31 December 2021, all the patients admitted to our emergency room (ER) with acute blunt or penetrating trauma were assessed for their injury pattern. Subsequently, the patients with liver injury confirmed via computer tomography (CT) were screened. A total of 26 patients who consented to blood sampling were assigned to two groups: injured patients with confirmed liver injury (AIS_Abdomen_ ≥ 2, *n* = 12) independently from Injury Severity Score (ISS), and polytraumatized patients with an ISS ≥ 16 in the absence of abdominal injury (AIS_Abdomen_ = 0, *n* = 14). All the patients included in the study were aged between 18 and 80 years. The patients who died in the ER or within 24 h of hospital admission, or had pre-existing immunological disorders, liver cirrhosis, benign and/or malignant liver tumors, chronic alcohol abuse, immunosuppressive and anti-coagulant medication, burns, concomitant acute myocardial infarction or thromboembolic events were excluded.

### 2.3. Study Design

All the trauma patients were treated according to the Advanced Trauma Life Support (ATLS) standards and current polytrauma management guidelines [23]. Injury severity was calculated using the Injury Severity Score (ISS) [24] and the New Injury Severity Score (NISS) [25] based on the Abbreviated Injury Scale (AIS) [26]. Demographic data (age and gender), injury severity parameters (ISS, NISS, AIS_Abdomen_), in-hospital Glasgow Coma Scale (GCS), pre- and in-hospital systolic blood pressure (SBP), heart rate (HR), amount of intravenously administered fluid, amount of packed red blood cells (PRBCs) administered within the initial 48 h after admission, length of stay at the intensive care unit (ICU), and in-hospital mortality were recorded.

Defined laboratory parameters (Interleukin (IL)-6 [pg/mL], leukocyte count [/nL], International Normalized Ratio (INR), activated Partial Thromboplastin Time (aPTT) [s], fibrinogen [mg/dL], platelet count [/nL], hemoglobin (Hb) [g/dL], albumin [g/dL], Aspartate Amino Transferase (ASAT, GOT) [U/L], Alanine Amino Transferase (ALAT, GPT) [U/L], Gamma Glutamyl Transferase (GGT) [U/L], and Base excess (BE) [mmol/L)]) were measured directly in the ER and on the first day (24 h) and second day (48 h) after admission.

### 2.4. Sample Collection

Blood samples (*n* = 26) were taken from the patients admitted to the ER of our level 1 trauma center within 6 h of trauma. The blood was collected in 7.5 mL/2.7 mL tubes (S-Monovette©, Sarstedt Inc., Nümbrecht, NRW, Germany) containing 1.6 mg EDTA K (plasma samples) or silicate-coated granules and polyacrylester gel (serum samples). Within 60 min, samples stored on ice were centrifuged for 15 min at 3500 revolutions per minute (rpm) and 4 °C. The supernatant was stored at −80 °C in a dedicated storage system until batch analysis [22].

### 2.5. microRNA Analysis

The extraction of miRNA was conducted using 200 µL of human plasma with the miRNeasy serum/plasma kit (Qiagen Inc., Hilden, Germany). The concentration of the isolated RNA was quantified photometrically using a spectrophotometer (Nanovue©, Harvard Bioscience Inc., Holliston, MA, USA). Reverse transcription (RT) was carried out with 20 ng miRNA using the miScript II RT kit (Qiagen). Polymerase chain reaction (PCR) was conducted following the manufacturer’s instructions by using the miScript SYBR^®^ Green PCR Kit (Qiagen). Amplification was performed using Stratagene Mx3005P QPCR Systems (Agilent Technologies Germany, Waldbronn, Germany) as follows: 1 cycle with 15 min at 95 °C, followed by 40 cycles with 15 s at 94 °C, 30 s at 55 °C, and 30 s at 70 °C, concluding with a dissociation curve. We used PCR assays (Qiagen) that comprised primers for the target miRNAs [hsa-miR-21-5p, -122-5p, -191-5p, -192-3p, and -212-3p (hsa: homo sapiens)] and the endogenous reference snoRNAs (hsa-SNORD38 and -SNORD48).

The selection of miRNAs for standardization is based on a previous study in which the homogeneous expression of both SNORD miRNAs was measured in polytrauma patients [27]. The mean value was calculated from the Ct values of both the standard miRNAs and the expression level of the target miRNAs was calculated using the delta Ct method [28].

### 2.6. L-FABP Analysis

Blinded specimens were used for duplicate measurements of L-FABP levels. Plasma L-FABP concentration was determined by using a commercially available highly specific ELISA (Hycult Biotechnology, Uden, The Netherlands) according to the manufacturer’s instructions.

### 2.7. Statistical Analysis

Statistical analyses and the presentation of the data as graphs were performed using GraphPad Prism 10 for Mac (Dotmatic, San Diego, CA, USA). The data are presented as mean ± standard deviation (SD). The *p*-values for continuous variables were calculated using the Mann–Whitney U test for two-group comparisons or the Kruskal–Wallis test for comparisons involving more than two groups. Significant values were adjusted using the Bonferroni post hoc test. Spearman’s rank correlation coefficients (r) were computed to assess the correlations between miRNA, laboratory parameters, and injury characteristics. To evaluate the diagnostic potential of the investigated biomarkers in detecting traumatic liver injury, receiver operating characteristic (ROC) curves were generated using simple and multiple logistic regression. The areas under the curves (AUC) with 95% confidence intervals (CIs) are presented. All the graphs are presented as scatter plots with the mean values displayed. Statistical significance was considered at * *p* ≤ 0.05, ** *p* ≤ 0.01, *** *p* ≤ 0.001, and **** *p* ≤ 0.0001.

## 3. Results

### 3.1. Demographics and Clinical Injury Characteristics

Table 1 shows the demographic and clinical characteristics stratified by injury pattern [liver trauma (LT) versus polytrauma without abdominal injury (PT)]. Twelve patients were included in the LT group and 14 patients in the PT group, totaling 26 trauma patients in this study. Most patients in both groups were male (LT group: 75%; PT group: 64.3%). The patients in the LT group tended to be younger than those in the PT group (*p* = 0.061). There was no significant difference between the two groups for either the ISS (*p* = 0.298) or the NISS (*p* = 0.144). The LT group had a significantly lower preclinical SBP value than the PT group (*p* = 0.027). Apart from this, there were no significant differences between the groups for other pre- and in-hospital parameters (see Table 1). The patients in the PT group tended to have longer stays in the intensive care unit (ICU) or Intermediate Care Unit (IMC) than those in the LT group (*p* = 0.054). All 26 patients survived and were discharged after completing the medical treatment (0% mortality in both groups).

### 3.2. Laboratory Parameters

#### 3.2.1. Systemic Profile of Inflammatory Marker Interleukin-6

**Figure 1:**
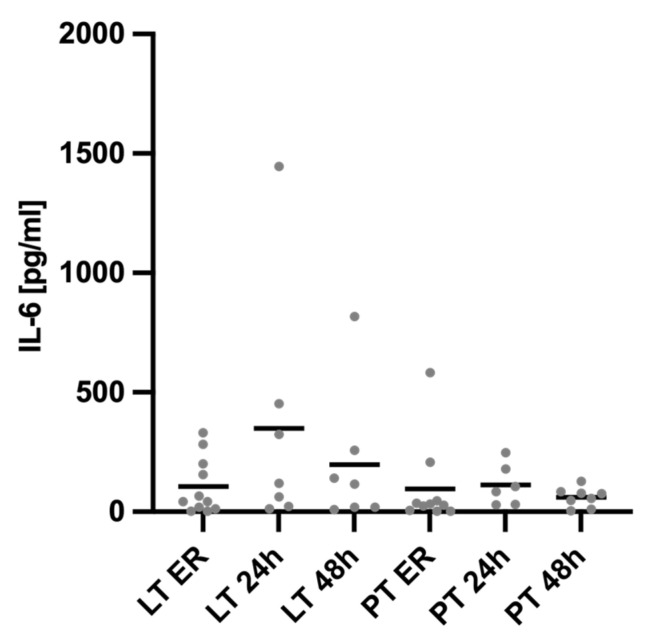
Plasma levels of Interleukin-6 (IL-6). There were no significant differences in the IL-6 levels between the groups and the respective time points. LT = liver trauma; PT = polytrauma.

Figure 1 shows the systemic Interleukin (IL)-6 levels over time, starting with admission to the ER and at 24 and 48 h after the trauma, stratified by injury pattern.

To investigate the potential impact of liver injury on the acute systemic inflammatory response, proinflammatory IL-6, which plays a central role in the acute phase response in the liver, was analyzed on admission to the ER and at 24 and 48 h post-trauma. Upon ER admission, the IL-6 levels were elevated in both groups, but the difference was not statistically significant (LT ER 105.1 ± 118.3 pg/mL vs. PT ER 96.53 ± 181.3 pg/mL, *p* = 0.524). A total of 24 h after trauma, both groups exhibited the highest IL-6 values measured, though the difference between groups remained non-significant (LT 24 h 348.4 ± 511.5 pg/mL vs. PT 24 h 196.7 ± 287.9 pg/mL, *p* = 0.731). By 48 h after trauma, the IL-6 values had decreased in both groups, with no significant difference between the LT- and PT-groups (LT 48 h 112.6 ± 86.44 pg/mL vs. PT 48 h 60.36 ± 40.10 pg/mL, *p* = 0.397).

#### 3.2.2. Systemic Profile of Coagulation Parameters

**Figure 2:**
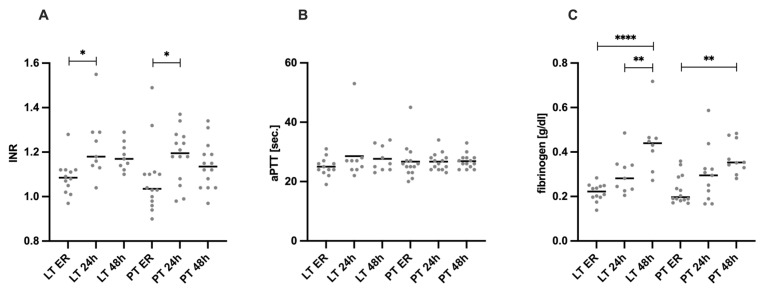
Plasma levels of coagulation parameters. (**A**) The International Normalized Ratio (INR) value is significantly lower in both the LT group and PT group at emergency room (ER) admission compared to the 24 h time point. (**B**) There was no significant difference between the groups with regard to the activated Partial Thromboplastin Time (aPTT). (**C**) Both the LT group and the PT group showed a significant increase in fibrinogen over the time points. * *p* ≤ 0.05, ** *p* ≤ 0.01 and **** *p* ≤ 0.0001. LT = liver trauma; PT = polytrauma.

Figure 2 depicts coagulation parameters (INR, aPTT, and fibrinogen) over time, starting with admission to the ER and at 24 and 48 h after trauma, stratified by injury pattern.

To investigate the possible influence of liver injuries on coagulation, typical coagulation parameters such as INR, aPTT, and fibrinogen were analyzed at admission to the ER and at 24 h and 48 h after trauma. In both the LT group (INR 1.09 ± 0.08) and the PT group (1.08 ± 0.16), a significant increase in the INR values was observed from trauma room admission to the 24 h time point (LT: 1.23 ± 0.15, *p* = 0.011; PT: 1.19 ± 0.12, *p* = 0.032).

The fibrinogen levels were within the normative range for both the LT group (ER 0.218 ± 0.040 g/dL) and the PT group (0.231 ± 0.064 g/dL, *p* = 0.752) at the time of ER (intrahospital reference range: 0.193–0.412 g/dL). Over the clinical course, the fibrinogen levels in the LT group increased significantly, reaching 0.299 ± 0.088 g/dL at the 24 h time point (LT ER vs. LT 24 h, *p* = 0.115) and further rising to 0.439 ± 0.133 g/dL by 48 h (LT ER vs. LT 48 h, *p* ≤ 0.0001). Similarly, the fibrinogen levels in the PT group also increased over time. From admission to the ER, the fibrinogen levels rose significantly to 0.298 ± 0.126 g/dL at 24 h (PT ER vs. PT 24 h, *p* = 0.73) and reached 0.375 ± 0.073 g/dL at 48 h (PT ER vs. PT 48 h, *p* = 0.002). There were no significant differences in aPTT between the groups and the respective time points.

#### 3.2.3. Systemic Profile of Liver Enzymes

**Figure 3:**
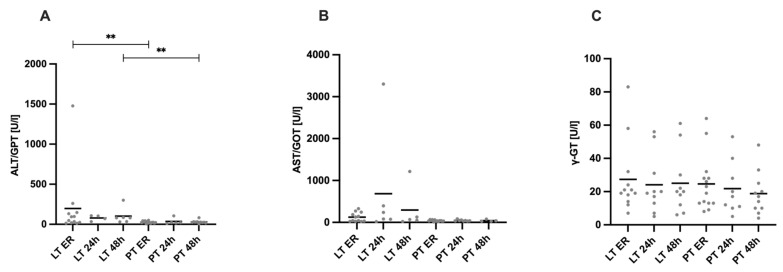
Plasma levels of liver enzymes. (**A**) The Alanine Aminotransferase (ALT/GPT) values were higher in the LT group than in the PT group, with significant differences between these two groups both at the time of admission to the ER and at 48 h after trauma. (**B**) There was a tendency towards higher Aspartate Aminotransferase (AST/GOT) values in the LT group than in the PT group at all the time points, with no significant differences. (**C**) There were no significant differences in the gamma-glutamyltransferase (γ-GT) levels between the groups and the respective time points. ** *p* ≤ 0.01. LT = liver trauma; PT = polytrauma.

Figure 3 shows liver enzymes (AST/GOT, ALT/GPT, and γ-GT) over time, starting with admission to the ER and at 24 and 48 h after the trauma, stratified by injury pattern.

Upon arrival in the ER, the AST levels in the LT group (124 ± 111.2 U/L) were higher than in the PT group (40 ± 17.01 U/L, *p* = 0.191). The AST levels remained higher in the LT group at 24 h (685.2 ± 1289 U/L vs. 46.63 ± 17.74 U/L in the PT group, *p* = 0.075) and 48 h (296 ± 515.3 U/L vs. 42.25 ± 23.60 U/L, *p* = 0.413) after trauma. Similarly, the ALT levels were significantly elevated in the LT group compared to the PT group on admission to the ER (196.8 ± 409.8 U/L vs. 26.29 ± 13.06 U/L, *p* = 0.05). The ALT levels remained higher in the LT group at 24 h (79 ± 34.51 U/L vs. 35.4 ± 39.75 U/L in the PT group, *p* = 0.1111) and were significantly elevated at 48 h (102.2 ± 100.4 U/L vs. 29.8 ± 20.16 U/L, *p* = 0.0075) after trauma. In contrast, no significant differences were observed between the LT and PT in the plasma levels of γ-GT across any of the time points analyzed.

### 3.3. Systemic Profile of miRNA Expression

**Figure 4:**
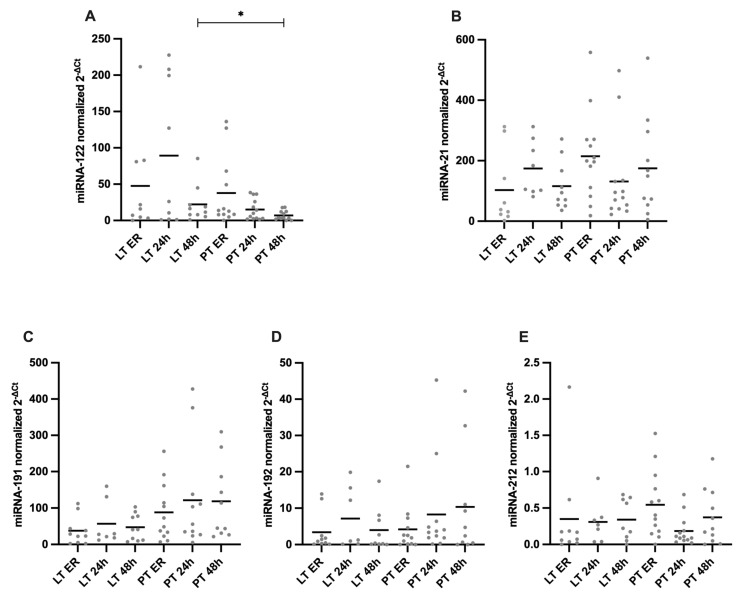
Plasma levels of different microRNAs (miRNA). (**A**) There was a tendency towards higher miRNA-122 values in the LT group than in the PT group at all the time points, with a significant difference between the 48 h time point in the LT group and the PT group. (**B**) There were no significant differences in the miRNA-21 levels between the groups and the respective time points. (**C**) There was a tendency towards higher miRNA-191 values in the PT group than in the LT group at all the time points, with no significant differences between the groups. (**D**,**E**) There were no significant differences in the miRNA-192 or miRNA-212 levels between the groups and the respective time points. * *p* ≤ 0.05. LT = liver trauma; PT = polytrauma.

Figure 4 shows the expression of various microRNAs (miRNA-122, miRNA-21, miRNA-191, miRNA-192, and miRNA-212) over time, starting with admission to the ER and at 24 and 48 h after the trauma, stratified by injury pattern.

In order to investigate whether liver-related miRNAs are increasingly released into the bloodstream during liver injury, the expression of specific miRNAs was analyzed upon admission to the ER, as well as at 24 and 48 h after trauma. The plasma levels of miRNA-122 tended to be higher in the LT group compared to the PT group at all the time points (LT ER 47.57 ± 69.47 vs. PT ER 37.79 ± 48.13, *p* = 0.862; LT 24 h 89.17 ± 100.1 vs. PT 24 h 15.14 ± 14.41, *p* = 0.471), with a significant difference observed at the 48 h time point (LT 48 h 22.09 ± 25.38 vs. PT 48 h 7.02 ± 6.6, *p* = 0.032). No significant differences in the miRNA-21 levels were observed between the groups at any time point (LT ER: 102.6 ± 121.9 vs. PT ER: 214.9 ± 145.9, *p* = 0.071; LT 24 h: 174 ± 90.44 vs. PT 24 h: 131.4 ± 148.6, *p* = 0.475; LT 48 h: 115.6 ± 80.75 vs. PT 48 h: 174.6 ± 161.4, *p* = 0.311). The miRNA-191 levels were consistently lower in the LT group compared to the PT group. On ER admission, the LT group had levels of 37.61 ± 38.59 compared to 88.26 ± 79.49 in the PT group (*p* = 0.081). This trend persisted at 24 h (LT 24 h: 57.02 ± 61.26 vs. PT 24 h: 121.6 ± 145.1, *p* = 0.285) and 48 h (LT 48 h: 47.23 ± 36.35 vs. PT 48 h: 118.7 ± 105.8, *p* = 0.058), though no significant differences were observed. There were no significant differences in the miRNA-192 levels between the two groups at the ER (LT ER 3.4 ± 5.25 vs. PT ER 4.18 ± 6.14, *p* = 0.722), 24 h (LT 24 h 7.16 ± 8.44 vs. PT 24 h 8.26 ± 13.39, *p* = 0.592), and 48 h (LT 48 h 3.96 ± 5.89 vs. 10.34 ± 14.97, *p* = 0.356) time points. Similarly, no significant differences were observed in the miRNA-212 levels between the groups at any time point (LT ER: 0.35 ± 0.66 vs. PT ER: 0.55 ± 0.46, *p* = 0.088; LT 24 h: 0.31 ± 0.29 vs. PT 24 h: 0.19 ± 0.2, *p* = 0.311; LT 48 h: 0.34 ± 0.28 vs. PT 48 h: 0.37 ± 0.38, *p* = 0.845).

### 3.4. Systemic Profile of L-FABP

**Figure 5:**
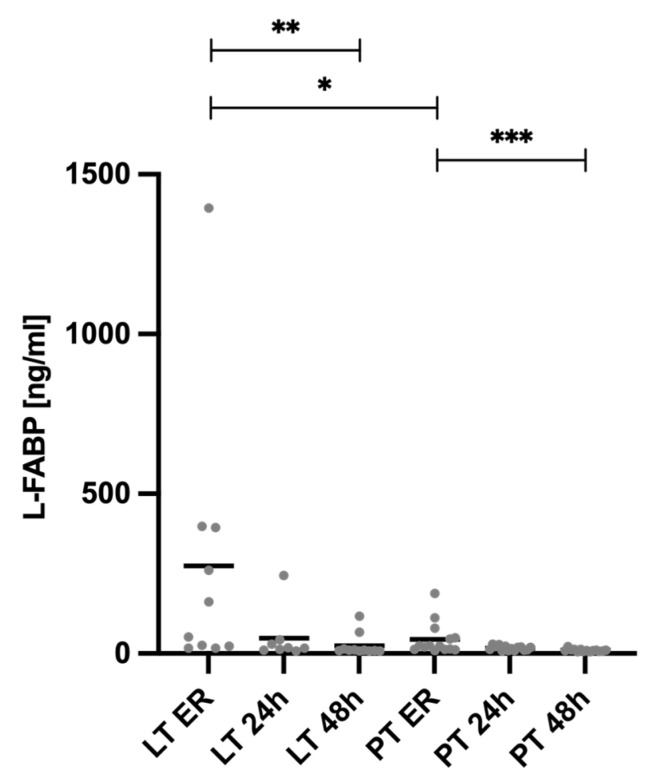
Plasma levels of liver fatty acid binding protein (L-FABP). The L-FABP value on admission to the emergency room (ER) was significantly higher in the LT group than in the PT group. In both groups, the L-FABP decreases significantly 48 h after trauma compared to the ER time point. * *p* ≤ 0.05, ** *p* ≤ 0.01, and *** *p* ≤ 0.001. LT = liver trauma; PT = polytrauma.

Figure 5 illustrates the systemic levels of the liver fatty acid binding protein (L-FABP) measured in the blood plasma over time, starting with admission to the ER and at 24 and 48 h after the trauma, stratified by injury pattern.

In addition to analyzing the miRNA expression, we also examined L-FABP to determine potential differences in protein expression between the two groups. Upon ER admission, the L-FABP levels in the LT group (274.2 ± 422.3 ng/mL) were significantly higher than those in the PT group (44.83 ± 50.64 ng/mL, *p* = 0.018). While the L-FABP levels remained higher in the LT group at 24 h (LT 24 h 48.2 ± 80.4 ng/mL vs. PT 24 h 17.2 ± 6.9 ng/mL, *p* = 0.525) and 48 h (LT 48 h 24.9 ± 34.7 ng/mL vs. PT 48 h 10.2 ± 4.2 ng/mL, *p* = 0.292) after trauma, these differences were not statistically significant. Furthermore, the L-FABP plasma levels in the LT group were significantly elevated at ER admission (274.2 ± 422.3 ng/mL) compared to 48 h post-trauma (24.9 ± 34.7 ng/mL, *p* ≤ 0.001). Similarly, in the PT group, the L-FABP levels at ER admission (44.83 ± 50.64 ng/mL) were significantly higher than those at 48 h (10.2 ± 4.2 ng/mL, *p* = 0.0002)

### 3.5. Correlations

In order to analyze the prognostic potential of the biomarkers L-FABP and miRNA-122, a correlation analysis with both the laboratory and clinical parameters was performed (Table 2). A strong correlation was found between L-FABP and the liver enzymes AST (rho = 0.788, *p* = 0.0056) and ALT (rho = 0.873, *p* = 0.0009) at the time of ER admission. Only a moderate correlation was observed for miRNA-122 with regard to AST (rho = 0.442, *p* = 0.174) and ALT (rho = 0.455, *p* = 0.164) for the same time point. For the severity of liver injury determined by AIS, L-FABP showed only a weak correlation (rho = 0.291, *p* = 0.384), while miRNA-122 showed a moderate correlation (rho = 0.599, *p* = 0.056) when being admitted to the ER.

Furthermore, the L-FABP levels at the ER correlated significantly with the length of stay in the ICU (rho = 0.763, *p* = 0.0086), whereas only a weak correlation was found for miRNA-122 and duration in the ICU (rho = 0.207, *p* = 0.539). There was a moderate correlation for the number of PRBCs transfused within the first 48 h after admission for both L-FABP (rho = 0.526, *p* = 0.109) and miRNA-122 (rho = 0.607, *p* = 0.055) upon ER arrival. Upon ER admission, we observed a strong correlation between L-FABP and miRNA-122 (rho = 0.673, *p* = 0.0277).

### 3.6. Simple and Multiple Logistic Regression

Using simple logistic regression, we examined to what extent the significantly elevated potential biomarkers (L-FABP in the ER, ALT in the ER, and miRNA-122 after 48 h) could predict the likelihood of indicating liver damage. For L-FABP at the ER time point, the AUC was 0.7792 (CI: 0.5934 to 0.9650; *p*-value = 0.0186). For ALT at the ER time point, the AUC was 0.7202 (CI: 0.5036 to 0.9369; *p*-value = 0.0570). For miRNA-122 after 48 h, the AUC was 0.7652 (CI: 0.5662 to 0.9641; *p*-value = 0.0312). The AUC for miRNA-122 at the ER time point was 0.5519 (CI: 0.3003 to 0.8036; *p*-value = 0.6614).

In the next step, multiple logistic regression was used to assess whether the combination of these potential biomarkers could indicate liver damage. The combination of ALT (ER), L-FABP (ER), and miRNA-122 (48 h) resulted in an AUC of 0.8712 (CI: 0.6904 to 1.000; *p*-value = 0.0026). The combination of ALT (ER) and L-FABP (ER) yielded an AUC of 0.7500 (CI: 0.5220 to 0.9780; *p*-value = 0.0423), while the combination of L-FABP (ER) and miRNA-122 (48 h) achieved an AUC of 0.7955 (CI: 0.6112 to 0.9797; *p*-value = 0.0164).

## 4. Discussion

In recent decades, the non-operative management (NOM) strategy has become the treatment of choice for hemodynamically stable patients with liver injury in the context of blunt abdominal trauma [29,30]. The safe use of this treatment regime is based on the development of standardized trauma care, the implementation of trauma centers, the use of ultrasound and CTs with steadily increasing resolution, the rapid availability of relevant laboratory parameters, changes in resuscitation protocols, and routine access to interventional procedures such as angiography and embolization [31,32,33,34]. The World Society of Emergency Surgery (WSES) guidelines for the treatment of liver trauma recommend serial clinical evaluations (physical exams, ultrasound, and laboratory testing) to detect a change in clinical status during NOM, with an emergency abdominal CT advised in the cases of clinical deterioration to identify the potential progression of the liver injury [35]. Common indicators of hepatic injuries after abdominal trauma are ALT and AST [36,37]. While elevated ALT levels were significantly higher in the LT group at admission and after 48 h compared to the PT group, AST levels, though elevated, were not statistically significant. Elevated AST values may also reflect damage to other tissues, such as skeletal muscle, rather than liver injury alone. Therefore, ALT is considered a more specific indicator of liver injury, though its predictive value remains inconsistent due to variability in cut-off values and ethnic differences [38,39,40,41].

Numerous studies have identified L-FABP as a reliable diagnostic marker for liver injury, superior to ALT and AST [42,43,44,45]. In this study, the L-FABP levels were significantly elevated in patients with liver upon admission, supporting its utility as a screening tool for liver-specific injuries. Similar findings were reported by Voth et al., who observed peak L-FABP levels in cases involving liver injury [11]. However, L-FABP is also expressed in other tissues, including skeletal muscle and kidneys, reducing its specificity in the context of multiple injuries. We found no strong correlation between the AIS_Abdomen_ scores and L-FABP levels, possibly due to the relatively low severity of liver injuries (average AIS_Abdomen_ of 2.5) and a smaller patient cohort compared to previous studies [10]. Recently, it was shown that the L-FABP levels were not only significantly elevated in isolated liver injuries, but also in kidney injuries and that these also correlated significantly with the AST, ALT, and creatinine values [12]. Both the LT and PT groups exhibited normal creatinine values, limiting conclusions about the impact of kidney injury on L-FABP levels. Further prospective studies are needed to clarify these findings.

In contrast to L-FABP, significant differences in the miRNA-122 levels between the LT and PT groups were observed only 48 h after trauma. Studies in mouse and rat models suggest that miRNA-122 plasma levels increase following liver damage (e.g., caused by acetaminophen, other chemicals, or specific diets) due to cellular release mechanisms, although the exact processes remain unclear [46,47]. Trauma-induced liver injury may exhibit a unique miRNA profile. For instance, Bala et al. showed that circulating miRNA-122 levels are compartment-specific depending on the type and severity of liver injury [48]. Additionally, recent studies in polytrauma models revealed dynamic changes in extracellular vesicle-packed miRNA-122, correlating with trauma severity [49]. The absence of significant miRNA-122 increases upon admission in this study may be attributed to the relatively mild liver injuries or differences in trauma severity compared to experimental models.

This study has limitations, notably the small sample size and the low severity of liver injuries in the cohort. Liver damage without concomitant injury to other abdominal organs is rare, limiting patient recruitment [50]. Additionally, the average AIS_Abdomen_ of 2.5 points likely reflects mild trauma, which may explain the lack of significant miRNA-122 increases. Future studies should incorporate larger cohorts, examine more severe liver injuries, and include a healthy control group to more effectively assess the potential of miRNAs as biomarkers. Moreover, the investigation of comprehensive miRNA panels through microarray or deep sequencing could enhance diagnostic accuracy. Studies have shown that miRNA panels are more sensitive and specific than single biomarkers in assessing liver injury and predicting outcomes [51,52].

## 5. Conclusions

Among the examined miRNAs, only miRNA-122 shows a modest but significant increase in traumatic liver injury compared to the non-liver injury trauma group, and only 48 h post-trauma. Traditional liver injury markers such as ALT and L-FABP already show significant differences upon admission, highlighting their clinical relevance. Future research with larger sample sizes should investigate whether a comprehensive panel of miRNAs could more accurately predict liver injury and the severity of hepatocellular damage while providing deeper insights into their role in regulating immunological processes during the acute post-traumatic phase.

## Figures and Tables

**Table 1:** Patients’ clinical data.

	Liver Trauma (*n* = 12)	Polytrauma (*n* = 14)	*p*-Value
Male [%]	9 (75%)	9 (64.3%)	0.683
Age [years] ± SD	35.3 ± 14.7	51.4 ± 22.1	0.061
ISS [points] ± SD	19.1 ± 11.8	23.3 ± 8.3	0.298
NISS [points] ± SD	22.8 ± 14.4	30.8 ± 12.5	0.144
AIS_Abdomen_ [points] ± SD	2.5 ± 0.8	0	**<0.0001**
SBP_pre-hospital_ [mmHg] ± SD	117 ± 23.3	141.6 ± 29.1	**0.027**
HR_pre-hospital_ [/minute] ± SD	95.2 ± 20.7	91.0 ± 20.0	0.607
Cristalloids_pre-hospital_ [mL] ± SD	883 ± 522	982 ± 639	0.901
GCS_ER_ [points] ± SD	9 ± 6.3	10.5 ± 5.9	0.692
SBP_ER_ [mmHg] ± SD	135 ± 36.2	149 ± 28	0.262
HR_ER_ [per minute] ± SD	87.7 ± 17.2	86.7 ± 25.6	0.914
Cristalloids_ER_ [mL] ± SD	1488 ± 2379	946.4 ± 867	0.726
Hemoglobin_ER_ [g/dL] ± SD	13.2 ± 2.2	13.2 ± 1.8	0.933
Base Excess_ER_ [mmol/L] ± SD	−3.6 ± 5.1	−2.1 ± 2.7	0.336
PRBCs_first 24 h_ [number a 330 mL] ± SD	0.4 ± 0.9	0.7 ± 1.3	0.571
Time on ICU/IMC [days] ± SD	7.1 ± 7.4	12.9 ± 11.5	0.054
Non-survivors [%]	0	0	

Abbreviations: dl: deciliter; g: gram; ER: emergency room; GCS: Glasgow Coma Scale; HR: heart rate; ICU: intensive care unit; IMC: Intermediate Care; ISS: Injury Severity Score; L: liter; mL: milliliter; mmHg: millimeter mercury; mmol: millimole; NISS: New Injury Severity Score; PRBC: packed red blood cell; SBP: systolic blood pressure; SD: standard deviation; The *p*-values below or equal to 0.05 are marked in bold.

**Table 2:** Correlation analysis of L-FABP and miRNA-122 with laboratory and clinical parameters.

Biomarker	Spearman Corr.	AST	ALT	AIS_Abdomen_	ICU Stay	PRBCs 48 h
L-FABP	Rho	0.788	0.873	0.291	0.763	0.526
	*p*-value	**0.0056**	**0.0009**	0.384	**0.0086**	0.109
miRNA-122	Rho	0.442	0.455	0.599	0.207	0.607
	*p*-value	0.174	0.164	0.056	0.539	0.055

Abbreviations: AIS: Abbreviated Injury Scale, AST: Aspartate Aminotransferase, ALT: Alanine Aminotransferase, Corr.: correlation, ICU: intensive care unit, PRBCs 48 h: Packed Red Blood Cells within 48 h after admission. The *p*-values below or equal to 0.05 are marked in bold.

## Data Availability

The original contributions presented in the study are included in the article. Further inquiries can be directed to the corresponding author.
